# Correction: Danzero et al. An Automated Cartridge-Based Microfluidic System for Real-Time Quantification of *BCR::ABL1* Transcripts in Chronic Myeloid Leukemia: An Italian Experience. *Int. J. Mol. Sci.* 2025, *26*, 8932

**DOI:** 10.3390/ijms27062784

**Published:** 2026-03-19

**Authors:** Alice Costanza Danzero, Enrico Marco Gottardi, Fabrizio Quarantelli, Ciro Del Prete, Alessandra Potenza, Claudia Venturi, Paola Berchialla, Francesca Guerrini, Clara Bono, Emanuela Ottaviani, Sara Galimberti, Carmen Fava, Barbara Izzo

**Affiliations:** 1 Department of Clinical and Biological Sciences, Turin University, 10043 Orbassano, Italy; enricogottardi@libero.it (E.M.G.); paola.berchialla@unito.it (P.B.); carmen.fava@unito.it (C.F.); 2 Oncologic Hematology and Cytogenetics Laboratory, CEINGE Advanced Biotechnology “Franco Salvatore”, 80131 Naples, Italy; quarantelli@ceinge.unina.it (F.Q.); delpreteci@ceinge.unina.it (C.D.P.); potenza@ceinge.unina.it (A.P.); 3 “Seràgnoli” Institute of Hematology, IRCCS University Hospital of Bologna, 40138 Bologna, Italy; ventclod85@gmail.com (C.V.); emanuela.ottaviani@aosp.bo.it (E.O.); 4 Department of Clinical and Experimental Medicine, Hematology, University of Pisa, 56126 Pisa, Italy; guerrinifra@libero.it (F.G.); sara.galimberti@unipi.it (S.G.); 5 Department of Molecular Medicine and Medical Biotechnology, University of Naples “Federico II” and CEINGE Advanced Biotechnology “Franco Salvatore”, 80131 Naples, Italy

In the original publication [1], the information related to the Institutional Review Board Statement and Informed Consent Statement was incorrectly stated. The correct Institutional Review Board Statement and Informed Consent Statement are provided below.

**Institutional Review Board Statement:** The study was conducted in accordance with the Declaration of Helsinki, and approved by the Ethics Committee of AOU San Luigi Gonzaga (166/2021, 22 July 2021).
**Informed Consent Statement:** Informed consent was obtained from all subjects involved in the study.

The authors state that the scientific conclusions are unaffected. This correction was approved by the Academic Editor. The original publication has also been updated.
