# Supplementary material for: An Automated Cartridge-Based Microfluidic System for Real-Time Quantification of BCR::ABL1 Transcripts in Chronic Myeloid Leukemia: An Italian Experience
Source: Int J Mol Sci. 2025 Sep 13;26(18):8932. doi: 10.3390/ijms26188932 (PMC12470050; doi:10.3390/ijms26188932)
Supplement: Supplementary file 1 [file ijms-26-08932-s001.zip › ijms-3766645-supplementary.pdf]

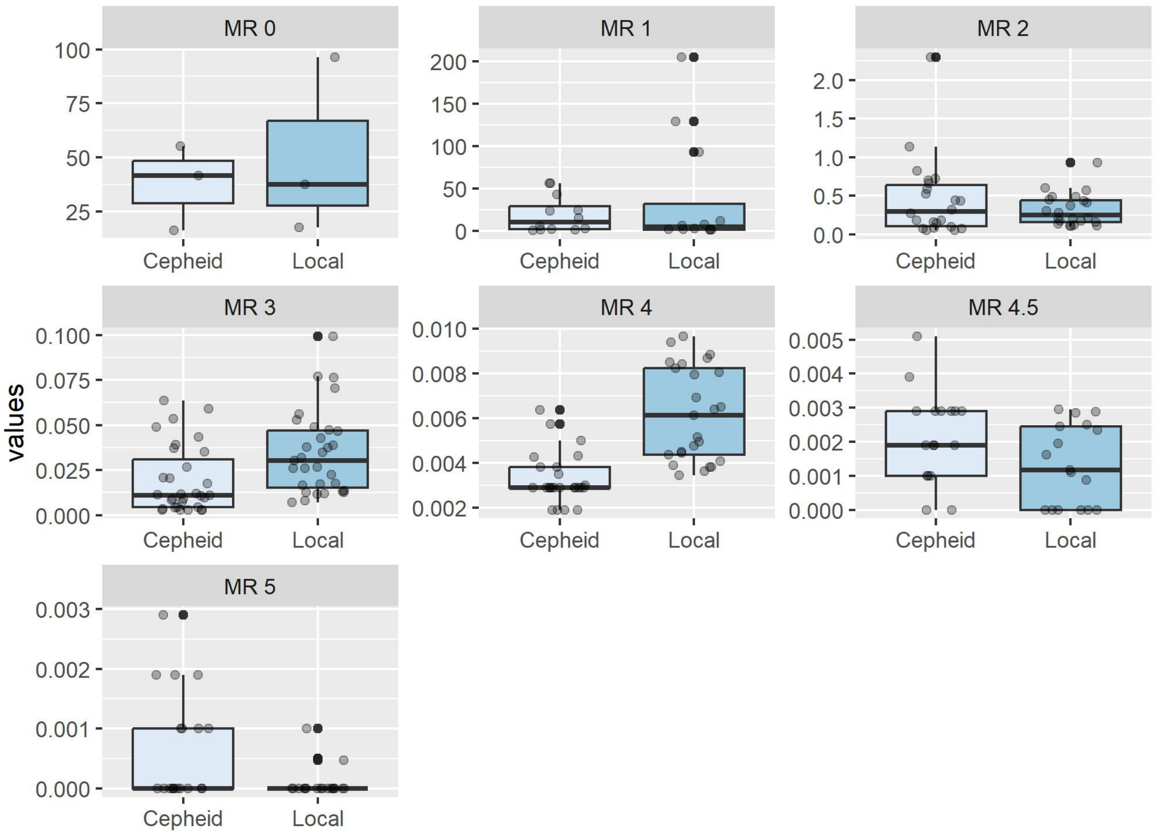

Supplementary Figure S1. Distribution of BCR::ABL1%IS by MR and Method (Cepheid vs Local).

Storage conditions for 24 hours	Number of samples	MR Mean ± SD Time 0	MR Mean ± SD Time +24h	Deviation from Time 0 %
**Lysate stored at -20°C.**	23	4,21 ± 0,87	4,23 ± 0,85	0,47
**Whole blood at room temperature (RT)**	14	3,42 ± 1,21	3,23 ± 1,17	-5,55
**Whole blood at 4°C**	14	4,36 ± 0,43	4,28 ± 0,51	-1,78

Supplementary Table S1. MR (mean ± SD) from Xpert® BCR-ABL Ultra at 0 h and 24 h under different storage conditions.

MR (mean ± SD) from Xpert® BCR-ABL Ultra analyses at Time 0 and after 24 h under different storage conditions: whole blood at room temperature (RT) or 4 °C, and lysate at −20 °C. % Deviation from Time 0 is reported to highlight changes over 24 h.

	Reference value %	Average of Cepheid values %	Deviation from Reference %
**AcroMetrix S1 (MR1, 10%)**	9.3	14.2217	+52,9
**AcroMetrix S2 (MR2: 1%)**	0.96	0.9475	-1,3
**AcroMetrix S3 (MR3: 0.1%)**	0.083	0.0604	-27,2
**AcroMetrix S4 (MR4: 0.01%)**	0.0071	0.0070	-1,4
**AcroMetrix S5 (MR4.5: 0.0032%)**	0.003	0.0029	-3,3
**UK NEQAS S1**	0.027	0.0179	-33,7
**UK NEQAS S2**	0.1	0.0925	-7,5

Supplementary Table S2. Comparison of AcroMetrix™ BCR-ABL Panel and UK NEQAS control panel values: reference versus Cepheid method.

The deviation from the reference value (% Deviation from Reference) was calculated as (Vm−Vr)/Vr×100, where Vm (measured value) is the Average of Cepheid values % and Vr (reference value) is the Reference value %.
